# Congenital Dyserythropoietic Anemia Type II: molecular analysis and expression of the *SEC23B *Gene

**DOI:** 10.1186/1750-1172-6-89

**Published:** 2011-12-30

**Authors:** Francesca Punzo, Aida M Bertoli-Avella, Saverio Scianguetta, Fulvio Della Ragione, Maddalena Casale, Luisa Ronzoni, Maria D Cappellini, Gianluca Forni, Ben A Oostra, Silverio Perrotta

**Affiliations:** 1 Department of Clinical Genetics, Erasmus Medical Centre, Rotterdam, the Netherlands; 2 Department of Paediatrics, Second University of Naples, Naples, Italy; 3 Department of Biochemistry and Biophysics, "F. Cedrangolo," Second University of Naples, Naples, Italy; 4 Department of Internal Medicine, Policlinic Foundation IRCCS, Milan University, Milan, Italy; 5 Centro della Microcitemia e Anemie Congenite, Galliera Hospital, Genova, Italy

**Keywords:** Congenital dyserythropoietic anemia, CDA II, SEC23B, Red blood cell, Coat complex protein II

## Abstract

**Background:**

Congenital dyserythropoietic anemia type II (CDAII), the most common form of CDA, is an autosomal recessive condition. CDAII diagnosis is based on invasive, expensive, and time consuming tests that are available only in specialized laboratories. The recent identification of *SEC23B *mutations as the cause of CDAII opens new possibilities for the molecular diagnosis of the disease. The aim of this study was to characterize molecular genomic *SEC23B *defects in 16 unrelated patients affected by CDAII and correlate the identified genetic alterations with *SEC23B *transcript and protein levels in erythroid precursors.

**Methods:**

*SEC23B *was sequenced in 16 patients, their relatives and 100 control participants. *SEC23B *transcript level were studied by quantitative PCR (qPCR) in peripheral erythroid precursors and lymphocytes from the patients and healthy control participants. Sec23B protein content was analyzed by immunoblotting in samples of erythroblast cells from CDAII patients and healthy controls.

**Results:**

All of the investigated cases carried *SEC23B *mutations on both alleles, with the exception of two patients in which a single heterozygous mutation was found. We identified 15 different *SEC23B *mutations, of which four represent novel mutations: p.Gln214Stop, p.Thr485Ala, p.Val637Gly, and p.Ser727Phe. The CDAII patients exhibited a 40-60% decrease of *SEC23B *mRNA levels in erythroid precursors when compared with the corresponding cell type from healthy participants. The largest decrease was observed in compound heterozygote patients with missense/nonsense mutations. In three patients, Sec23B protein levels were evaluated in erythroid precursors and found to be strictly correlated with the reduction observed at the transcript level. We also demonstrate that Sec23B mRNA expression levels in lymphocytes and erythroblasts are similar.

**Conclusions:**

In this study, we identified four novel *SEC23B *mutations associated with CDAII disease. We also demonstrate that the genetic alteration results in a significant decrease of *SEC23B *transcript in erythroid precursors. Similar down-regulation was observed in peripheral lymphocytes, suggesting that the use of these cells might be sufficient in the identification of Sec23B gene alterations. Finally, we demonstrate that decreased Sec23B protein levels in erythroid precursors correlate with down-regulation of the *SEC23B *mRNA transcript.

## Background

Congenital dyserythropoietic anemias (CDAs) are a group of rare hereditary disorders characterized by ineffective erythropoiesis and distinct morphological abnormalities of the erythroblasts in the bone marrow [1]. CDA type II (CDAII, OMIM 224100), which is transmitted as an autosomal recessive condition, is the most frequent; the main European Registries (German, Italian and French) have counted 367 patients [2]. The clinical picture is characterized by mild to moderate anemia associated with jaundice, splenomegaly, and iron overload [3,4]. In clinical practice, evidence of CDAII is primarily based on bone marrow examination [5,6]. Confirmation of diagnosis is based on at least one of the following biochemical tests, including: a positive acid serum lysis test with ABO-compatible sera; band 3 protein glycosylation defects evidenced by sodium dodecyl sulphate-polyacrylamide gel electrophoresis (SDS-PAGE); a discontinuous double membrane in mature erythroblasts (visible by electron microscopy), and the presence of endoplasmic reticulum (ER)-specific proteins [5,7-9]. However, these tests are expensive, time consuming, and often available in only a few specialized laboratories. For these reasons, the correct diagnosis of CDAII is often delayed or erroneously suspected.

A major breakthrough in CDAII research was achieved in 2009, when Schwarz et al. and Bianchi et al. found mutations of the *SEC23B *gene in patients with CDAII [10,11]. Sec23B protein is an essential component of coat protein complex II (COPII), coated vesicles that transport secretory proteins from the ER to the Golgi complex [12]. So far, *SEC23B *changes have been identified mainly by direct genomic sequencing of the coding region of the gene [10,11,13-15]; however, the precise effects of the described mutations on the RNA expression level in erythroid cells has not been studied. Moreover, a reduction of Sec23B protein in CDAII erythroid precursors has not been reported.

In this study, we investigated *SEC23B *gene mutations, by both genomic and cDNA direct sequencing, in 16 unrelated Italian CDAII patients from 16 families. In all cases, we identified *SEC23B *mutations, and four of these were novel. We also evaluated the effects of different *SEC23B *mutations on mRNA and protein expression levels.

## Methods

### Patients

We collected blood samples from 16 unrelated Italian CDAII patients belonging to 16 families and 100 unrelated Italian controls (included in the DNA sequence analyses). The diagnosis of CDAII was made on the basis of clinical features, bone marrow examination, and/or SDS-PAGE. All patients provided their written informed consent for the study, which was approved by the research ethics committee of the Second University of Naples, Italy. The study was conducted in accordance with the Declaration of Helsinki.

### Erythroid precursor cultures

After informed consent had been obtained, peripheral blood from CDA II patients and from 5 healthy control relatives was collected into sterile heparinised tubes. Light-density mononuclear cells obtained by centrifugation on Lymphoprep (Nycomed Pharma) density gradient were enriched for CD34^+ ^cells by positive selection using CD34 microbeads (Miltenyi Biotech) according to the manufacturers' instructions. CD34^+ ^cells were cultured at a density of 10^5 ^cells/mL in alpha-minimal essential medium (α-MEM; GIBCO) supplemented with 30% fetal bovine serum (FBS; GIBCO), as previously described [16]. To induce cells proliferation and erythroid differentiation, cells were cultured with 20 ng/mL rH stem cell factor (SCF, PeproTech), 10 ng/mL rH interleukin-3 (IL-3, PeproTech) and 3 U/mL recombinant human (rH) erythropoietin (rHuepo, Janssen-Cilag). Cells were incubated at 37°C with an atmosphere of 5% CO_2 _for 14 days; after 7 days of culture the medium was changed to ensure good cells feeding. Cell samples were collected on days 14 of culture (mature erythroblast stage) for further analysis.

### Molecular analysis of the SEC23B gene

Genomic DNA was isolated using the Flexigene DNA extraction kit (Qiagen). All *SEC23B *exons, their flanking splice junctions, and their 5'- and 3'-untranslated regions were amplified with 21 polymerase chain reactions (PCRs). cDNA was prepared, using the iScript cDNA synthesis kit (Bio-Rad), from approximately 100 ng mRNA obtained from lymphocytes (Trizol Reagent Kit - Invitrogen) from all 16 patients and 8 healthy control relatives. cDNA was obtained also from erythroblasts [16] from 8 patients (ID: F1, G2, B3, A4, C5, B11P13, and M15) and 5 of the 8 healthy control relatives mentioned above. The coding region of the *SEC23B *cDNA was covered by six PCR fragments. Sequences of all primers can be found in Table 1. The PCR conditions were: 94°C for 5 min; 30 cycles of 94°C for 30 sec, 58°C for 30 sec and 72°C for 30 sec; and 72°C for 7 min. Amplified DNA and cDNA were purified (Exo-Sap-IT) and sequenced using BDT v3.1 on an ABIPrism 3130XL genetic analyzer. Sequences were analyzed using the SeqScape program, version 2.6 (Applied Biosystems).

**Table 1:** Primer sequences for SEC23B cDNA amplification

Oligo Name	Oligo Sequence
SEC23B cDNA 1F	ACCTGTCTTGCCCTGTTCC
SEC23B cDNA 1R	TACAGGCCCAAAGTTTTGCT
SEC23B cDNA 2F	AGCAGGCCAACTTGTAAAGC
SEC23B cDNA 2R	CTTGAAGCAAAAGGGTGCTC
SEC23B cDNA 3F	ACAGGATATGTTGGGCCTGA
SEC23B cDNA 3R	TTGCACAACACTTCATCTCCA
SEC23B cDNA 4F	GAACAGCTGCAAATGGTCAC
SEC23B cDNA 4R	CACAGTCGGATGAGTTGTCG
SEC23B cDNA 5F	GACCGACAACTCATCCGACT
SEC23B cDNA 5R	TTTCCTGTCCCCAAGCATAC
SEC23B cDNA 6F	CAGTCAGGCTCGATTCCTTT
SEC23B cDNA 6R	CACCTAAACAAGCTGCCAAA

### Real-time PCR

cDNA was prepared from patients' mRNA from lymphocytes and erythroblasts. Real-time PCR was performed in accordance with manufacturers' instructions. The reactions were run on an ABI 7300 real-time PCR system (Applied Biosystems); the cycling conditions were 10 min at 95°C (initial denaturation) followed by 40 cycles of 15 sec at 94°C (denaturation) and 1 min at 68°C (annealing/extension/data collection). In the first step, we determined the stability of a control gene (β-actin) for the normalization of the real-time PCR products. The linearity and efficiency of this assay were tested over dilutions of input cDNA spanning five orders of magnitude. Assays were performed in triplicate. We used the 2^-ΔΔCt ^method to analyze the data obtained.

### Western blotting

Proteins were extracted from erythroid cultures of patients A4, B3, and C5 using RIPA Lysis Buffer (Millipore) and following the manufacturer's instructions. Sec23B was characterized in total lysates from erythroid cultures by Western blotting. Membranes were incubated overnight at 4°C with rabbit polyclonal anti-Sec23B antibody (1:500 dilution; SAB2102104, Sigma-Aldrich); reactive bands were detected by chemiluminescence (SuperSignal). An anti-β-actin antibody (1:500 dilution; Sigma) was used to check for comparable protein loading and as a housekeeping protein. Images were captured, stored, and analyzed using Quantity One software (BioRad).

## Results

### Molecular analysis of the SEC23B gene

We identified *SEC23B *mutations in all 16 patients enrolled in the study. Among the 15 mutations characterized, four are novel: c.640C > T, c.1453A > G, c.1910T > G, and c.2180C > T (Table 2). In total, we identified 10 missense, three nonsense, one in-frame deletion of 3 nucleotides, and one splice-site mutation. The splice-site alteration creates a new donor site after exon 2 of the *SEC23B *gene. Most of the investigated patients were compound heterozygotes. Only four patients had homozygous *SEC23B *mutations; three of these were homozygotes for the c.325G > A variant that leads to the amino acid change p.Glu109Lys (the most frequent *SEC23B *mutation encountered, with a prevalence of 32% among CDAII patients). The fourth patient was homozygous for the c.1254T > G mutation (p.Ile418Met).

**Table 2:** SEC23B mutations in 16 Italian patients

Patient ID	Allele 1	Allele 2	Protein change 1	Protein change 2	cDNA %
F1	c.953 T > C	**c.1910 T > G**	p.Ile318Thr	p.Val637Gly	55
G2	c.40 C > T	c.1015 C > T	p.Arg14Trp	p.Arg339X	42
B3	c.325 G > A	c.325 G > A	p.Glu109Lys	p.Glu109Lys	60
A4	IVS1 +31 A > G	c.367 C > T	Donor site ins	p.Arg123X	36
C5	c.40 C > T	c.1857-1859delCAT	p.Arg14Trp	p.I619del	54
C6	c.40 C > T	c.2101 C > T	p.Arg14Trp	p.Arg701Cys	62
P7	c.40 C > T	c.1015 C > T	p.Arg14Trp	p.Arg339X	42
C9	c.40 C > T	-	p.Arg14Trp	-	58
D10	c.325 G > A	c.325 G > A	p.Glu109Lys	p.Glu109Lys	60
B11	**c.1453 A > G**	c.1589 G > A	p.Thr485Ala	p.Arg530Gln	55
P13	c.40 C > T	**c.640 C > T**	p.Arg14Trp	p.Gln214X	50
G14	c.325 G > A	c.325 G > A	p.Glu109Lys	p.Glu109Lys	61
M15	c.40 C > T	**c.2180 C > T**	p.Arg14Trp	p.Ser727Phe	55
F16	c.325 G > A	c716 A > G	p.Glu109Lys	p.Asp239Gly	61
P17	c.40 C > T	-	p.Arg14Trp	-	64
E18	c.1254 T > G	c.1254 T > G	p.Ile418Met	p.Ile418Met	53

Novel mutations are bold

In two patients, we observed only a single heterozygous mutation (Table 2). Because individuals with a heterozygous *SEC23B *mutation are not affected by CDAII, we investigated in detail the putative occurrence of heterozygous exon deletions/insertions in the alternative allele in these patients. PCR of cDNA using primers located in the 5' and 3' untranslated regions of the gene revealed full-length transcript in both patients and no aberrant products, excluding the presence of heterozygous exon deletions/insertions. cDNA sequence analysis confirmed the heterozygous missense mutation and the presence of two alleles (i.e., wild-type and mutant).

**Figure 1:**
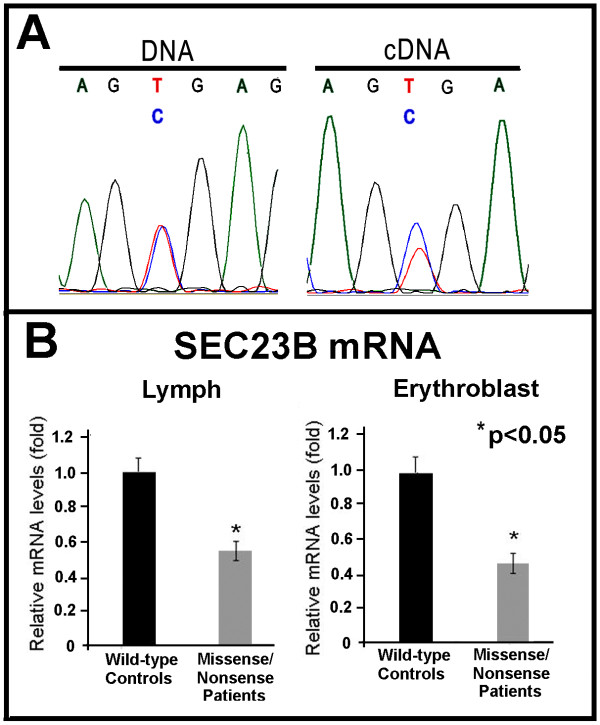
**A) Electropherogram depicting the nonsense mutation (c.367C > T) observed after DNA and cDNA sequencing; B) Relative *SEC23B *mRNA expression levels in lymphocytes and erythroblasts from patients with nonsense/missense mutations compared with healthy controls**.

We also sequenced *SEC23B *cDNA from the 16 CDAII patients. In all cases, the mutations identified by genomic analysis were confirmed by cDNA sequencing. Even in the patients carrying nonsense mutations (which most likely lead to RNA decay), it was possible to visualize the genetic change by cDNA sequencing (Figure 1A). None of the novel mutations described in this study was found in 100 unrelated Italian controls. All mutations were absent from the 1094 individuals from the 1000Genomes project.

### Real-time PCR

To directly evaluate the consequences of the genomic *SEC23B *mutations, we investigated *SEC23B *mRNA levels in patients and healthy individuals using quantitative PCR (qPCR). Patients that were compound heterozygotes for a missense and a nonsense mutation exhibited a drastic decrease in mRNA expression levels of approximately 50-60% when normalized to the endogenous control gene β-actin (Table 2 and Figure 1B). Patients with two missense mutations showed a milder reduction in *SEC23B *mRNA levels (approximately 40-45%) (Table 2). The results were similar when using RNA from lymphocytes or mature erythroblasts (Figure 1B and data not shown). The two patients with a single heterozygous mutation on *SEC23B *(C9 and P17) also exhibited a reduction of *SEC23B *transcript (Table 2).

**Figure 2:**
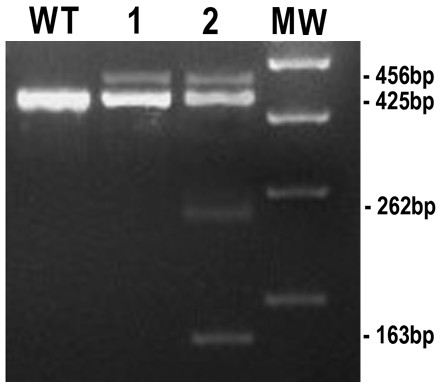
**Characterization of *SEC23B *mutations in patient A4**. Lymphocytic cDNA was amplified using primers localized in exons 1 and 4 (A4-SEC23B-1F: TTGTACCCCTGGCTTGTCTC and A4-SEC23B-4R: ATGAACCTGCACCATCCTTC). The control shows the 425-bp product (wild-type, W.T.); (1) Patient A4: Two bands (456-bp and 425-bp) were present. The additional 456-bp band is due to the splicing mutation: c.221+31 A > G; (2) The nonsense mutation (c.367 C > T) creates a restriction site for the enzyme HpyCH4III. *SEC23B *PCR product from patient A4 was enzymatically digested. The two resulting bands (262-bp and 163-bp) represent the cut allele carrying the nonsense mutation. M.W. = Molecular Weight.

Patient A4 was compound heterozygote for a splicing mutation (c.221+31A > G) and a nonsense change (c.367C > T). In this individual, we suspected very low or no wild-type (WT) transcript. Therefore, we investigated A4 *SEC23B *mRNA more in detail. First, the presence of the +31A > G allele was confirmed on agarose gel by the presence of an additional 31-bp band. In the other allele, the nonsense mutation creates a restriction site for the enzyme HpyCH4III. After enzymatic digestion of the PCR product with HpyCH4III restriction enzyme, we observed four fragments: an upper band for the + 31A < G allele, two lower bands representing the cut allele carrying the nonsense mutation, and a normal-sized band (Figure 2). The occurrence of a normal transcript indicates that at least a small amount of WT *SEC23B *RNA is present. This finding corresponds to the observed 35-40% *SEC23B *mRNA expression level measured by qPCR.

### Western blotting

**Figure 3:**
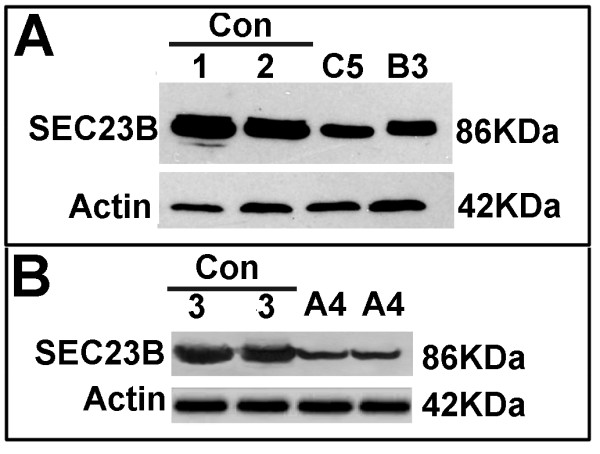
**Sec23B protein analysis in erythroid precursors**. Western blots from patients C5 and B3 (panel A), and A4 (panel B) demonstrate reduced Sec23B expression compared with control participants (Con). Approximately 40 μg of protein were loaded. β-actin was used as a loading control. The experiment was representative of two different experiments.

Finally, we investigated the amount of Sec23B protein in the erythroblasts of 3 CDAII patients (C5, B3 and A4) by immunoblotting. The Sec23B content was normalized to β-actin. As depicted in Figure 3A, the Sec23B content of patients C5 and B3 was clearly reduced compared to two different controls. Similar results were obtained in two independent experiments. Moreover, the estimated Sec23B protein level in erythroblasts from patient A4 suggested that it corresponded to approximately 30-35% of that of a healthy individual (Figure 3B). Therefore, our data suggest good correspondence between the transcript amount and protein content, underscoring the usefulness of mRNA evaluation.

## Discussion

In this study, we identified four novel *SEC23B *gene mutations by analyzing 16 Italian patients with CDAII (Table 2). We also identified two CDAII patients with only one heterozygous mutation each.

Since the initial identification of *SEC23B *mutations in CDAII patients, 59 mutations have been identified, including the current work (Table 3) [10,11,13-15,17,18]. Two missense mutations have been repeatedly identified in a large proportion of patients: p.Glu109Lys and p.Arg14Trp (32% and 19%, respectively). To date, these missense mutations account for approximately 50% of the mutant alleles in CDAII patients (Table 3). Notably, evidence of a founder effect has been described for p.Glu109Lys among Israeli Moroccan Jewish patients [13]. Concerning mutation type, missense (52%) and nonsense (21%) mutations are the most commonly observed followed by deletions or insertions that lead to frameshifts in the nucleotide sequence. Splicing mutations are rare, with only six mutations reported (Table 3).

**Table 3:** Summary of all SEC23B mutations in CDAII patients, predicted effect on protein and allelic frequencies

Exon/Intron	Nucleotide change	Protein change	Type of mutation	Allelic frequency, n (%)	References
2	c.40 C > T	p.Arg14Trp	Missense	48 (19)	[11]
2-3	c.221+31 A > G	-	Splice-site change*	2 (0.8)	[14]
2	c.53 G > A	p.Arg18His	Missense	3 (1.2)	[11]
2	c.197 G > A	p.Cys66Tyr	Missense	1 (0.4)	[17]
3	c.235 C > T	p.Arg79X	Non-sense	3 (1.2)	[11]
3-4	c.279 +3 A > G	-	Splice-site change	1 (0.4)	[14]
3-4	c.222-817_366+4242del	-	Frame-shift	1 (0.4)	[11]
4	c.325 G > A	p.Glu109Lys	Missense	81 (32)	[11]
5	c.367 C > T	p.Arg123X	Non-sense	2 (0.8)	[14]
5	c.387(delG)	p.Leu129LeufsX26	Frame-shift	1 (0.4)	[14]
5	c.428delAinsCG	-	Frame-shift	1 (0.4)	[10]
5	c.568 C > T	p.Arg190X	Non-sense	1 (0.4)	[10]
6	c.640 C > T	p.Gln214X	Non-sense	1 (0.4)	Present study
6	c.649 C > T	p.Arg217X	Non-sense	4 (1.6)	[10]
6-7	c.689+1G > A	-	Splice-site change	4 (1.6)	[10]
7	c.716 A > G	p.Asp239Gly	Missense	3 (1.2)	[11]
7	c.790 C > T	p.Arg264X	Non-sense	3 (1.2)	[11]
8	c.938 G > A	p.Arg313His	Missense	4 (1.6)	[11]
8	c.953 T > C	p.Ile318Thr	Missense	6 (2.4)	[11]
8	c.970 C > T	p.Arg324X	Non-sense	2 (0.8)	[11]
9	c.1015 C > T	p.Arg339X	Non-sense	3 (1.2)	[14]
9	c.1043 A > C	p.Asp348Ala	Missense	1 (0.4)	[10]
9	c.1063delG	-	Frame-shift	1 (0.4)	[11]
9-10	c.1109 +5 G > A	-	Splice-site change	1 (0.4)	[14]
9-10	c.1190 +1 G > A	-	Splice-site change	1 (0.4)	[14]
10	c.1157 A > T	p.Gln353Leu	Missense	1 (0.4)	[11]
10	c.1201 C > T	p.Arg401X	Non-sense	1 (0.4)	[11]
11	c.1254 T > G	p.Ile418Met	Missense	3 (1.2)	[14]
11	c.1276 G > A	p.V426I Poly	Missense	2 (0.8)	[11]
11	c.1307 C > T	p.Ser436Leu	Missense	1 (0.4)	[14]
12	c.1385 A > G	p.Tyr462Cys	Missense	6 (2.4)	[11]
13	c.1453 A > G	p.Thr485Ala	Missense	1 (0.4)	Present study
13	c.1489 C > T	p.Arg497Cys	Missense	9 (3.6)	[10]
13	c.1508 G > A	p.Arg503Gln	Missense	1 (0.4)	[18]
14	c.1571 C > T	p.Ala524Val	Missense	5 (2)	[11]
14	c.1588 C > T	p.Arg530Trp	Missense	1 (0.4)	[11]
14	c.1589 G > A	p.Arg530Gln	Missense	2 (0.8)	[18]
14	c.1603 C > T	p.Arg535X	Non-sense	2 (0.8)	[14]
14	c.1648 C > T	p.Arg550X	Non-sense	4 (1.6)	[18]
14	c.1654 C > T	p.Leu552Phe	Missense	1 (0.4)	[14]
14	c.1660 C > T	p.Arg554X	Non-sense	2 (0.8)	[10]
15	c.1685 A > G	p.Tyr562Cys	Missense	1 (0.4)	[18]
15	c.1733 T > C	p.Leu578Pro	Missense	2 (0.8)	[14]
15	c.1735 T > A	p.Tyr579Asn	Missense	1 (0.4)	[14]
16	c.1808 C > T	p.Ser603Leu	Missense	1 (0.4)	[10]
16	c.1821delT	-	Frame-shift	3 (1.2)	[10]
16	c.1832 G > C	p.Arg611Pro	Missense	1 (0.4)	[14]
16	c.1858 A > G	p.Met620Val	Missense	2 (0.8)	[14]
16	c.1857_1859delCAT	p.Ile619del	In frame deletion**	2 (0.8)	[14]
17	c.1910 T > G	p.Val637Gly	Missense	1 (0.4)	Present study
17	c.1962-64delT	p.Thr654ThrfsX13	Frame-shift	1 (0.4)	[18]
17	c.1968 T > G	p.Phe656Leu	Missense	1 (0.4)	[18]
18	c.2101 C > T	p.Arg701Cys	Missense	9 (3.6)	[10]
18	c.2129 C > T	p.Thr710Met	Missense	1 (0.4)	[13]
18-19	c.2149 -2 A > G	-	Splice-site change	2 (0.8)	[14]
19	c.2150(delC)	p.Ala717ValfsX7	Frame-shift	1 (0.4)	[14]
19	c.2166 A > C	p.Lys723Gln	Missense	1 (0.4)	[18]
19	c.2180 C > T	p.Ser727Phe	Missense	1 (0.4)	Present study
20	c.2270 A > C	p.His757Pro	Missense	1 (0.4)	[14]

*creation of a new donor site

**1 aminoacid deletion

The *SEC23B *gene appears to play a pivotal and probably unique function in erythroid precursors [19,20]. Although detailed genetic analyses have been conducted, the effects of the mutations on mRNA content in erythroblast cells have not been documented. Moreover, no data are available about the effects of mutations on Sec23B protein content in red cell precursors.

To evaluate the effect of missense and nonsense mutations on *SEC23B *mRNA expression levels, we performed quantitative (qPCR) analysis of *SEC23B *transcripts on all of our patients. In this study, we used both cDNAs prepared from erythroid precursor cultures and peripheral lymphocytes. Although we demonstrate that all patients have a significant reduction of *SEC23B *mRNA, this reduction was more pronounced in patients with missense/nonsense mutations (Table 2). From a diagnostic point of view, it is interesting to note that the results obtained in erythroid precursors and lymphocytes were comparable, suggesting that peripheral lymphocytes not only represent a good source of *SEC23B *transcript, but also replicate the effect of the genetic change observable in the erythroid population.

In addition, to search for genotype-phenotype correlation, we grouped patients according to their degree of anemia. We did not observe any correlation between degree of anemia, type of mutation, and relative *SEC23B *mRNA reduction (data not shown).

Almost all CDAII patients harbor mutations in both *SEC23B *alleles. In a few cases (10 out of 111 described in the literature, or 9%), only a single heterozygous *SEC23B *mutation has been found. This finding raises the possibility of the occurrence of mutations that have thus far escaped the exon screening technology. In our study, two CDAII patients were identified in whom a mutation was observed in only one allele. In these participants, mRNA analysis (cDNA sequencing and long-range PCR on cDNA) confirmed the presence of both wild-type and mutated alleles. However, qPCR analysis revealed a reduction in *SEC23B *mRNA expression of approximately 40% in these patients compared with control participants, similar to the reduction observed in patients with two missense mutations. This finding suggests the possible occurrence of mutations that affect the regulatory regions of the *SEC23B *gene. Alternate mechanisms such as microRNA dysregulation could be responsible for CDAII in these cases where the second heterozygote mutation has not been found.

Here we demonstrate that *SEC23B *mutations result in reductions of both the relative transcript and protein content in erythoid precursors. So far, only one study has investigated protein levels of Sec23B in CDAII patients [11]. No reduction of Sec23B protein levels was observed, most likely due to the type of cell used in the study (fibroblasts). Our data, although comprising a small number of cases, clearly demonstrate that CDAII erythroblastoid cells show a strong reduction of the protein that parallels the data regarding mRNA levels. Future investigations are necessary to clarify the effect of protein reduction on the patients' phenotype.

Patients lacking Sec23B expression have never been described. We identified a single patient (A4) with a nonsense mutation and a splice site mutation (c.221+31 A > G) that causes a stop codon after exon 1. In our view, this patient could have had a very strong reduction of Sec23B expression. On the basis of this hypothesis, we analyzed the amount of *SEC23B *mRNA and protein in this patient in detail. The results demonstrate that there is still a small amount of WT *SEC23B *mRNA and that the Sec23B protein level in this patient corresponds to 30% of the level observed in healthy participants, suggesting that the absence of *SEC23B *expression may be lethal.

## Conclusions

This study reports *SEC23B *gene mutations in all 16 CDAII patients studied, confirming the causative relevance of the gene to the condition. We also demonstrated that the *SEC23B *gene mutations lead to a remarkable reduction of *SEC23B *transcript in erythroid precursors, the cell type altered in the disease. We also demonstrated that quantifying and sequencing *SEC23B *mRNA from peripheral lymphocytes (and not only from erythroid cultures) might facilitate the genetic diagnosis of CDAII. Our data on heterozygote patients suggest (although indirectly) the occurrence of rare mutations is not restricted to its coding regions. Finally, we demonstrate that the relative mRNA reduction directly corresponds to a protein decrease in erythroblastoid cells. Future studies will be devoted to characterizing the effect of SEC23B protein down-regulation on erythropoiesis and clarifying *SEC23B *gene regulation.

## List of abbreviations

bp: Base pair; COPII: Coat Protein Complex II; CDAs: Congenital Dyserythropoietic Anemias; CDAII: Congenital Dyserythropoietic Anemia type II; ER: Endoplasmic reticulum; PCR: Polymerase Chain Reaction; qPCR: Quantitative PCR; rH: Recombinant human; SDS-PAGE: Sodium Dodecyl Sulphate-Polyacrylamide Gel Electrophoresis; WT: Wild-type.

## Competing interests

The authors declare that they have no competing interests

## Authors' contributions

FP wrote the paper, performed the experiments, and analyzed data. AMB-A. contributed to the writing of the paper and study design. SS performed experiments and analyzed data. MC contributed to patient selection and clinical characterization. LR and MDC produced erythroid precursor cell lines. GLF and AI contributed to patient selection and clinical characterization. BAO and FDR contributed to study design. SP designed the research study, selected patients, and contributed to the writing of the paper. All authors read and approved the final manuscript.
